# The relevance of theobromine for the beneficial effects of cocoa consumption

**DOI:** 10.3389/fphar.2015.00030

**Published:** 2015-02-20

**Authors:** Eva Martínez-Pinilla, Ainhoa Oñatibia-Astibia, Rafael Franco

**Affiliations:** ^1^Laboratory of Cell and Molecular Neuropharmacology, Department of Neuroscience, Center for Applied Medical Research, University of Navarra, Pamplona, Navarra, Spain; ^2^Official College of Pharmacists of Gipuzkoa, San Sebastián, Spain; ^3^Molecular Neurobiology Laboratory, Department of Biochemistry and Molecular Biology, Faculty of Biology, University of Barcelona, Barcelona, Spain

**Keywords:** caffeine, theobromine, cocoa, adenosine receptor, neurological disease, receptor antagonist

## Abstract

Cocoa consumption began in America and in the mid sixteenth Century it quickly spread to Europe. Beyond being considered a pleasant habit due to its rich sweet lingering taste, chocolate was considered a good nutrient and even a medicine. Traditionally, health benefits of cocoa have been related with the high content of antioxidants of *Theobroma cocoa* beans. However, the direct psychoactive effect due to methylxanthines in cocoa is notable. Theobromine and caffeine, in the proportions found in cocoa, are responsible for the liking of the food/beverage. These compounds influence in a positive way our moods and our state of alertness. Theobromine, which is found in higher amounts than caffeine, seems to be behind several effects attributed to cocoa intake. The main mechanisms of action are inhibition of phosphodiesterases and blockade of adenosine receptors. Further mechanisms are being explored to better understand the health benefits associated to theobromine consumption. Unlike what happens in other mammals -pets- included, theobromine is safe for humans and has fewer unwanted effects than caffeine. Therefore, theobromine deserves attention as one of the most attractive molecules in cocoa.

## INTRODUCTION

**FIGURE 1:**
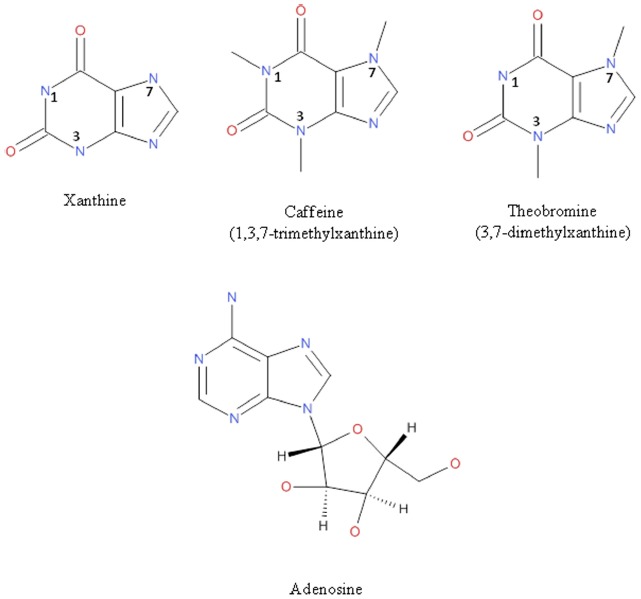
**Chemical structure of xanthine, caffeine, theobromine, and adenosine**.

Caffeine present in coffee and in cola beverages is heavily consumed worldwide. The reason of such high consumption relates to its benefits for day-life activities. Caffeine actions in the central nervous system (CNS) are fundamental to understand the interest of the intake of caffeine-containing beverages. Beneficial actions range from alertness to reducing the risk of neurodegenerative diseases. Although the highest concentration of caffeine is present in coffee, cocoa also contains this methylxanthine (Figure 1) but at doses that probably are not enough to activate neural mechanisms. However, cocoa has elevated concentrations of a structurally similar component, theobromine. The effects of theobromine have been less studied than those of caffeine but it is known that this molecule exerts some positive effects in different human pathologies. The combination of caffeine and theobromine in cocoa may have the expected methylxanthine-derived benefits without the side effects reported for caffeine. Interestingly, the main action mechanism of caffeine and theobromine consists of blocking adenosine receptors and inhibiting phosphodiesterases. The present paper takes data of novel studies that point toward alternative modes of action of theobromine. Further research is, however, required to fully understand the health benefits of cocoa consumption.

## THEOBROMINE AND CAFFEINE CONTENT IN COCOA

The physiological effects of cocoa components and theobromine in particular, deserve to be closely scrutinized to better understand the properties of cocoa consumption. The differences between coffee and cocoa perceived by consumers are mainly due to their most abundant molecules: caffeine in coffee and theobromine in cocoa. Moreover, the high contents of carbohydrates in cocoa products may be a further factor to consider.

Besides the cocoa proven psychoactive potential, caffeine and theobromine content is in full or in part responsible for the liking of this food. Human volunteers consuming a drink plus a capsule containing the two compounds, in amounts equivalent to those found in 50 g of dark chocolate (19 mg caffeine and 250 mg theobromine), liked the drink more than when the pairing was with a capsule containing placebo (Smit and Blackburn, 2005). These results, probably mediated by adenosine receptors, are conclusive of reinforcing actions of methylxanthines at doses and proportions found in cocoa. It is important to note that neither caffeine nor theobromine are addictive substances (see National Institute on Drug Abuse, 2014) and also they are not in the list of doping substances provided by the World Anti-Doping Agency (see The World Anti-Doping Agency (WADA), 2014).

## THEOBROMINE STUDIES IN MAMMALS: SAFETY AND TOXICITY

*In vivo* effects of xenobiotic or synthetic drugs require the use of animal models. However, theobromine, appears to be toxic in some mammals, including pets (Smit, 2011). Laboratory animal toxicity is a factor to consider in the extrapolation of data to humans. Reasons for this toxicity are not well established but unequivocally suggest that the action mechanisms of theobromine in humans may be different from those observed in other mammals. Due to these facts, the molecular pharmacology of theobromine, in particular its effect on adenosine receptors must be revisited using human tissue samples and cells, or heterologous systems expressing human proteins. The knowledge of adverse effects in some animals has probably prompted a relatively high number of clinical trials that prove that theobromine is not toxic for humans (Pendleton et al., 2012, 2013; Baggott et al., 2013) but has benefits in a variety of conditions (see below). It should be noted that the link between cocoa consumption and risk of preeclampsia in pregnant women, described previously, has not been proven. However, recent systematic reviews suggest the benefits of cocoa intake in the prevention of gestational hypertension (Klebanoff et al., 2009; Mogollon et al., 2013).

## CAFFEINE, THEOBROMINE, AND ADENOSINE RECEPTORS

The main pharmacological effects of caffeine, largely due to its structural similarity to adenosine molecule (Figure 1), include the inhibition of phosphodiesterases (enzymes that degrade the second messenger, cAMP), the regulation of intracellular calcium levels and the antagonism of adenosine receptors (Choi et al., 1988; McPherson et al., 1991; Chen and Chern, 2011; Johnson et al., 2012; Tazzeo et al., 2012). These primary actions result in the well-described physiological effects of caffeine as stimulant of CNS (Smit et al., 2004; Ciruela et al., 2006). Moreover, this methylxanthine can also perform other peripheral processes such as relax smooth muscles or stimulate the diuresis and cardiac muscle contraction (Tazzeo et al., 2012). Caffeine is mainly metabolized by the liver and, interestingly, one of its metabolites is theobromine (Becker et al., 1984).

As methylxanthines, caffeine and theobromine (Figure 1), are blockers of adenosine receptors which are G-protein-coupled receptors that sense the presence of extracellular adenosine. Adenosine is both an intermediate metabolite and also a messenger molecule that exerts its hormone-like action in the periphery and acts as a potent neuroregulator in the CNS. Four receptor subtypes for the compound have been identified: A_1_, A_2A_, A_2B_, and A_3_, widely distributed in the human body although with differential cell/tissue expression. Brain physiology is dependent upon variations in the concentration of adenosine that impacts on adenosine receptors in neurons. In this sense, a quick way to start the daily activities is disrupting the effect of adenosine in the brain by using blockers of its specific receptors. Technically such blockers are called “antagonists” and, therefore, caffeine and theobromine are antagonists of adenosine receptors. Growing evidence in the last decade indicates that theobromine has psychoactive actions in humans that are qualitatively different from those of caffeine (Mitchell et al., 2011; Baggott et al., 2013). The effect of theobromine on blood pressure (van den Bogaard et al., 2010) is also qualitatively different than that of caffeine (Mitchell et al., 2011) but the reasons for these differences are not established.

One possible explanation for the discrepancy in the effects of caffeine and theobromine could be their different half-life. Half-life of theobromine is higher than caffeine even in rodents, which have a faster hepatic metabolism. Thus, half of the theobromine administered to rats is excreted unchanged (Bonati et al., 1984). The mean half-life in plasma from healthy volunteers is approximately 10 h and the percentage of unmodified compound present in urine collected for 48 h after a single dose of 10 mg/Kg is relatively high (16–18% depending on the technique for isolation and quantitation; Tarka et al., 1983). The importance of this fact is evidenced when methylxanthines are used as bronchodilators in the management of asthma patients in whose serum the half-life is also higher for theobromine than for caffeine (Becker et al., 1984). When one of the main xenobiotic metabolizing enzymes, cytochrome P450 1A2 (YP1A2), is expressed in heterologous cells the rate of transformation is much lower for theobromine (5%) than for caffeine (81%; Gu et al., 1992) thus confirming that caffeine is more labile in terms of degradation than theobromine. Effects of *in vivo* administration of caffeine are in part due to the products of its metabolism. As relatively stable compound, theobromine may play a crucial role in some beneficial effects attributed to caffeine.

Theobromine is useful in asthma and in other respiratory tract problems such as cough for which no definitive drug has been developed. Codeine is very effective but its metabolism to compounds acting on opioid receptors limits its use (Prieto-Lastra et al., 2006). A safety and natural alternative could be theobromine since it is able to prevent cough provoked by citric acid in guinea-pigs and by capsaicin (an irritant component of chili peppers) in humans. This double-blind placebo-controlled study was complemented with *in vitro* studies using human *vagus* nerve preparations in which theobromine inhibited the depolarization effect of capsaicin (Usmani et al., 2005). Bearing in mind these results, theobromine seems to suppress cough by inhibiting the activation of afferent nerves. Two clinical trials have been completed to test antitussive action of theobromine but no results are available yet. In one of them (NCT01416480 identifier in clinicaltrials.org) 300 mg of theobromine capsules were used for antitussive effects in acute bronchitis. In a second study (NCT01656668 identifier in clinicaltrials.org) capsules of 300 mg theobromine were evaluated in frequent long-term cough. Whether cocoa consumption may be helpful to prevent coughing or to diminish cough intensity remain to be determined.

Noteworthy, van Zyl et al. (2008) reported that the diffusion of theobromine in lung substructures is higher than that of other drugs used in the therapy of respiratory diseases. The authors suggest that not only lipophilicity but also the position of alkyl groups in the purine ring affect the ability of caffeine and theobromine to cross biological membranes. The differential capability of tissue penetration and accumulation may explain why theobromine may achieve higher effects than caffeine. Although theobromine may have less affinity for receptors than caffeine, the efficacy of theobromine may become higher if it readily crosses membranes and reaches high interstitial concentrations.

Benefits of the theobromine on cough seem to be related with its anti-inflammatory potential as well as with modulation of airway reactivity (Mokry et al., 2009). Non-selective phosphodiesterase inhibitors are already efficacious in suppression of airway hyperreactivity. From the dozen existing enzymes cleaving cyclic mononucleotides (cAMP/cGMP), phosphodiesterase four is a good choice as therapeutic target in cough suppression (Mokry and Nosalova, 2011). Cortijo et al. (1993) showed an enrichment of phosphodiesterase four in human bronchial tissue and a good correlation between enzyme inhibition and bronchorelaxation potency. Recently, Sugimoto et al. (1994, 2014) have demonstrated that the antitumor potential effect of theobromine in malignant glioblastoma proliferation results from regulation of phosphodiesterase four, protein kinase B, extracellular signal-regulated p38 mitogen-activated protein kinase and nuclear factor-kappa B. Acting as phosphodiesterase inhibitors, methylxanthines are able to downregulate pro-inflammatory cytokines such as interferon-gamma or tumor necrosis factor-alpha (Harris et al., 2010). Apart from a direct theobromine effect on phosphodiesterases, the results are consistent with blockade of adenosine receptors negatively affecting adenylate cyclase activity, i.e., those coupled to G_i_ proteins (A_1_ and A_3_ receptors).

## ADENOSINE RECEPTOR-INDEPENDENT EFFECTS OF THEOBROMINE

Despite mainly acting as adenosine antagonist, theobromine may have actions that are not mediated by the blockade of these receptors. Theobromine and other main components of a hydro-alcoholic guaraná extract are able to reduce cell toxicity caused by nitric oxide generation (Bittencourt et al., 2013). It is unlikely that reduction of oxidative stress, DNA damage and lipid peroxidation in cells by the guaraná extract are mediated by blocking adenosine receptors.

In recent years, theobromine is starting to be widely studied to look for common and differential mechanisms with caffeine. Theobromine and caffeine are methylxanthines that may form non-covalent stacking complexes with ATP (Gattuso et al., 2011) and affect cell metabolism and/or DNA and RNA structure (Johnson et al., 2012). In fact, theobromine and caffeine are able to bind to DNA at millimolar concentrations (Johnson et al., 2012) and theobromine can also interact with RNA (Johnson et al., 2003). However, the full physiological consequences of these findings are not known yet. One hypothesis proposes that sustained interaction with DNA and RNA after consumption of methylxanthines in cocoa, might lead to induce or repress gene expression. Oleaga et al. (2012) have shown that a polyphenolic extract of cocoa alters the expression of genes in human breast cancer cells. Accordingly, one attractive possibility is the impact in the expression of genes with potential to decrease the risk of neurodegenerative diseases. Recent reports indicated that chronic consumption of coffee leads to reduced risk of Alzheimer’s and of Parkinson’s disease (Maia and de Mendonça, 2002; Costa et al., 2010; Eskelinen et al., 2011; Messerli, 2012). This beneficial effect is totally linked to a continued consumption at mid life, i.e. intake of methylxanthine-containing products reduces neurodegeneration later in life (Pelligrino et al., 2010; Klaassen et al., 2013; Haller et al., 2014).

The effect of theobromine in respiratory diseases is not due to inhibition of mediators of inflammation in asthma, histamine or slow reacting substance of anaphylaxis (Hillyard et al., 1984). A novel differential target of methylxanthines is poly(ADP-ribose)polymerase-1, a nuclear enzyme that is poorly inhibited by caffeine but significantly inhibited by theobromine (Geraets et al., 2006). In this sense, Ahmad et al. (2015) have recently shown that inhibition of poly(ADP-ribose)polymerase-1 significantly reduces inflammation of lungs caused by gamma-carrageenan. Recent evidence demonstrates neovascularization in an animal model of asthma (Wagner et al., 2015). Interestingly, theobromine may reduce neovascularization accompanying tumor growth and metastasis (Gil et al., 1993) and, therefore, it may reduce both acute symptoms and angiogenesis in asthma.

Exposure to nitrogen mustards causes lung inflammation and upregulation of oxidative stress proteins in macrophages. The analog of theobromine, pentoxifylline, is effective in reducing inflammation and increasing the number of macrophages with wound repair anti-inflammatory properties (Sunil et al., 2014). The concentration of adenosine at inflammation sites is notable (Cronstein et al., 1999) and, consequently, it can activate adenosine receptors present in lung cells and in macrophages. Blockade of adenosine receptors and/or inhibition of phosphodiesterases may underlie the phenotypic changes caused by methylxanthines in macrophages activated after the mustard inhalation.

A pilot study was developed to test whether theobromine was able to protect the enamel surface of human molars. The results of this *in vitro* study showed that two different concentrations of theobromine were able to preserve the structure of the teeth treated three days with acidic hydroxyl-ethyl-cellulose for demineralization (Kargul et al., 2012). This protective effect may not be due to adenosine receptors since they are not present on enamel surfaces. Theobromine benefits at this level were attained at relatively high concentrations. Actually, cocoa contains carbohydrates that may be metabolized by bacteria in the mouth and causing dental caries so caution may be taken to consider cocoa intake as protector for teeth. Sugar-free cocoa alternative could result in benefits to reduce caloric intake and preventing dental caries.

Other adenosine receptor-independent effect of theobromine is demonstrated in cardiovascular protection by significant increases in HDL cholesterol plasma levels and decreases in LDL ones. Clinical trials have been undertaken in volunteers taking cocoa to assess the effect of this substance on plasma lipoprotein levels (Kris-Etherton et al., 1994; Mursu et al., 2004; Wang-Polagruto et al., 2006; Baba et al., 2007; Mellor et al., 2010; Khan et al., 2012). The results of the clinical trial NCT01481389 (clinicaltrials.org) suggest that theobromine but not flavonoids is the responsible for the increase in HDL levels in individuals taking cocoa products (Neufingerl et al., 2013). The mechanism of HDL-increasing effect is probably multifactorial and non-necessarily related to the blockade of adenosine receptors. Likely based on a diuretic effect in dogs (Macnider, 1917), theobromine has been considered useful for weight loss and it is supplemented to herbal tea preparations (Khazan et al., 2014). However, there is neither enough data to confirm weight-loss potential in humans nor the putative underlying mechanism.

## CONCLUSION

Over the last decades, a remarkable progress has allowed understanding some of the molecular mechanisms that are behind the proved health benefits of cacao consumption in man. Apart from the high content of antioxidants, solid evidence points to methylxanthines as key players in the beneficial effects. Caffeine has been classically considered with higher potential than other methylxanthines. Recent studies have highlighted the potential of theobromine, which may act as antitumoral, anti-inflammatory or cardiovascular protector molecule without the undesirable side effects described for caffeine. The main mechanisms of action of theobromine are inhibition of phosphodiesterases and blockade of adenosine receptors but, interestingly, it exhibits other important adenosine receptor-independent effects as the reduction of cellular oxidative stress or regulation of gene expression. In this sense, theobromine could be considered a safe and natural alternative in the treatment of some human diseases and may serve as lead compound for the development of novel drugs

### Conflict of Interest Statement

The authors declare that the research was conducted in the absence of any commercial or financial relationships that could be construed as a potential conflict of interest.
